# Supplementary material for: The Systems Biology Research Tool: evolvable open-source software
Source: BMC Syst Biol. 2008 Jun 29;2:55. doi: 10.1186/1752-0509-2-55 (PMC2446383; doi:10.1186/1752-0509-2-55)
Supplement: Additional file 1 — SBRT Archive. An archive of the current version of the Systems Biology Research Tool. [file 1752-0509-2-55-S1.zip › sbrt-1.4.0/doc/developers_guide/api/sbrt/kernel/util/Or.html]

Or


|  |  |  |
| --- | --- | --- |
| **Overview** \| **Package** \| **Class** \| **Use** \| **Tree** \| **Deprecated** \| **Index** \| **Help** | |  |
| **PREV CLASS**   **NEXT CLASS** | **FRAMES**    **NO FRAMES**     **All Classes** |  |
| SUMMARY: NESTED \| FIELD \| CONSTR \| METHOD | DETAIL: FIELD \| CONSTR \| METHOD |  |


---


## sbrt.kernel.util Class Or<E>

```
java.lang.Object
  sbrt.kernel.util.BooleanRelation<E>
      sbrt.kernel.util.Or<E>
```

**All Implemented Interfaces:**: java.lang.Iterable<E>

---

``` public final class Or<E> extends BooleanRelation<E> ```

---

**Constructor Summary**

`Or(java.util.Collection<E> elements)`


| **Method Summary** | |
| --- | --- |
| `boolean` | `equals(java.lang.Object obj)` |
| `java.lang.String` | `toString()` |

**Methods inherited from class sbrt.kernel.util.BooleanRelation**

`contains, elements, getElements, hashCode, iterator`

**Methods inherited from class java.lang.Object**

`clone, finalize, getClass, notify, notifyAll, wait, wait, wait`

**Constructor Detail**

### Or

```
public Or(java.util.Collection<E> elements)
```


**Method Detail**

### equals

```
public boolean equals(java.lang.Object obj)
```

:   **Specified by:**: `equals` in class `BooleanRelation<E>`

---


### toString

```
public java.lang.String toString()
```

:   **Overrides:**: `toString` in class `java.lang.Object`


---


|  |  |  |
| --- | --- | --- |
| **Overview** \| **Package** \| **Class** \| **Use** \| **Tree** \| **Deprecated** \| **Index** \| **Help** | |  |
| **PREV CLASS**   **NEXT CLASS** | **FRAMES**    **NO FRAMES**     **All Classes** |  |
| SUMMARY: NESTED \| FIELD \| CONSTR \| METHOD | DETAIL: FIELD \| CONSTR \| METHOD |  |


---
